# AI-Assisted In-House Power Monitoring for the Detection of Cognitive Impairment in Older Adults

**DOI:** 10.3390/s21186249

**Published:** 2021-09-17

**Authors:** Yuriko Nakaoku, Soshiro Ogata, Shunsuke Murata, Makoto Nishimori, Masafumi Ihara, Koji Iihara, Misa Takegami, Kunihiro Nishimura

**Affiliations:** 1 Department of Preventive Medicine and Epidemiology, National Cerebral and Cardiovascular Center, Suita 564-8565, Japan; yurikon@ncvc.go.jp (Y.N.); s_ogata@ncvc.go.jp (S.O.); murata.s@ncvc.go.jp (S.M.); takegami@ncvc.go.jp (M.T.); 2 Division of Epidemiology, Kobe University Graduate School of Medicine, Kobe 650-0017, Japan; makoto84@med.kobe-u.ac.jp; 3 Department of Neurology, National Cerebral and Cardiovascular Center, Suita 564-8565, Japan; ihara@ncvc.go.jp; 4 National Cerebral and Cardiovascular Center, Suita 564-8565, Japan; kiihara@ncvc.go.jp

**Keywords:** power monitoring, in-house monitoring, cognitive impairment, dementia

## Abstract

In-home monitoring systems have been used to detect cognitive decline in older adults by allowing continuous monitoring of routine activities. In this study, we investigated whether unobtrusive in-house power monitoring technologies could be used to predict cognitive impairment. A total of 94 older adults aged ≥65 years were enrolled in this study. Generalized linear mixed models with subject-specific random intercepts were used to evaluate differences in the usage time of home appliances between people with and without cognitive impairment. Three independent power monitoring parameters representing activity behavior were found to be associated with cognitive impairment. Representative values of mean differences between those with cognitive impairment relative to those without were −13.5 min for induction heating in the spring, −1.80 min for microwave oven in the winter, and −0.82 h for air conditioner in the winter. We developed two prediction models for cognitive impairment, one with power monitoring data and the other without, and found that the former had better predictive ability (accuracy, 0.82; sensitivity, 0.48; specificity, 0.96) compared to the latter (accuracy, 0.76; sensitivity, 0.30; specificity, 0.95). In summary, in-house power monitoring technologies can be used to detect cognitive impairment.

## 1. Introduction

The number of people living with dementia is projected to triple by 2050 to 150 million, as the world’s aging population continues to grow [1]. Mild cognitive impairment (MCI) has been regarded as a prodrome of dementia, involving a decline in certain objective cognitive functions yet preserving independence in everyday life and not meeting the criteria for dementia [2,3]. Thus, in order to prevent dementia, the consensus is that primary interventions should be focused on this MCI population. However, the prevalence of MCI is difficult to ascertain due to differences in definitions and methods used in research, resulting in a wide range of estimates (3–42%) of people aged ≥65 years [4].

The detection of cognitive impairment is often delayed, and many people with the disorder remain undiagnosed. There are a number of possible reasons for this. First, screening tests for cognitive impairment are performed at clinics and hospitals, but people with poor subjective symptoms rarely go to hospitals for screening. Second, a complete neuropsychological assessment requires information gathering from cohabiting family members who are aware of the patient’s daily life. Yet, as many patients live alone in this aging society, it is difficult to obtain a reliable, comprehensive clinical picture of the patient’s real-world status. Thus, sensitive and objective measures are crucial to aid with early detection of cognitive decline.

As a solution to these current real-world difficulties in timely detection of cognitive impairment, we propose the use of in-house power monitoring to identify people with cognitive impairment. Power monitoring falls into the category of in-home monitoring in a broad sense, providing the capability to automatically collect information on everyday behaviors without imposing any restrictions on people’s routines. Advantages of in-home monitoring are objective, long-term follow-up with continuous assessment that helps to avoid some measurement biases [5]. Further, this continuous assessment can capture intraindividual variability that may be the earliest indicator of meaningful changes in routine daily activities [6,7]. Identifying individuals with cognitive impairment could lead to targeted dementia interventions that ultimately improve the daily function and independence of patients.

According to a systematic review of in-home monitoring sensor technologies for cognitive impairment detection, a number of studies have identified early signs of dementia from smart home-based behavioral data [8]. However, as conventional in-home monitoring systems require sensors to be installed in many rooms, there are potential obstacles to implementing these technologies in real life situations (e.g., installation, frequent maintenance, and privacy concerns). In this study, we used an unobtrusive (non-wearable and non-camera) in-house power monitoring technology developed by Informetis Co., Ltd., Tokyo, Japan, referred to as non-intrusive load monitoring (NILM) technology. It is a device separation estimation technology that can be used to grasp life patterns based on the usage of in-home electric appliances regardless of the manufacturer or model of the home appliance, with a single sensor attached to the power distribution board in each home. The present study is the first to use this in-house power monitoring system to predict dementia.

The present study aimed to evaluate differences in the usage time of home appliances between Japanese older adults with and without cognitive impairment, and to develop a preliminary prediction model for cognitive impairment by using in-house power monitoring data.

## 2. Related Work

There has been no report of a model that actually predicts cognitive impairment using in-house power disaggregation technology (including NILM technology), along with its prediction performance. A large number of reports have been published on the methods and infrastructure of Ambient Assisted Living (AAL) and electric power disaggregation technology, which are described below.

AAL is an emerging field of research using intelligent and pervasive computing technologies, which mainly focuses on supporting older people to live safely and autonomously in their home environment [9]. Globally, there is a growing demand for enhancing the health of older people via the use of technology [10,11]. A survey by Grguric et al. [11] showed the AAL models and architectures dealt with heterogeneous data sources and data flow; the survey confirmed no commonly accepted standard of detection by sensors. This study analyzed and compared previous works and architectures, which included various functions, such as general health monitoring, wandering prevention tools, and fall detection systems. In Europe, Nikoloudakis et al. [12] reported that the AAL European Programme aims to foster emergency alert systems for wandering based on the AAL system. The author showed that the utilization of AAL systems can allow caretakers to constantly monitor a patient’s indoor/outdoor position and receive notification when they leave predefined safety area [12].

Stavropoulos et al. [13] developed the DemaWare2 framework, which integrates a wide range of sensor modalities and technologies, together with semantic fusion of audio analysis techniques and plugs in the context of AAL. Sensors included not only ambient and wearable devices, but also plugs in smart spaces. As a result, the average duration of daily activity tasks including use of kettles, cups, and smartphones made it possible to distinguish between people with Alzheimer’s disease and those without.

Naeem et al. [14] discussed an overview of the current methods for unobtrusively recognizing activities of daily living (ADL) within a home environment for people with physical or cognitive disabilities. The study proposed a detailed overview of feature detection and accurate activity recognition of ADL decline using in-home sensors.

Calmers et al. [15] demonstrated how the analysis of electricity usage through load disaggregation can be used to model behavioral routines. Machine learning algorithms sufficiently identified five appliances, including the kettle, microwave, toaster, electric oven, and washing machine. Results from the clinical trial showed that important ADLs can be detected and used to facilitate behavioral analysis, using these five devices alone. In this paper, the system was tested in a clinical trial with two people with dementia over a 6-month period. This study showed that in-house power monitoring system could monitor dementia patients and detect the disease progression at home.

Many machine learning approaches to appliance load monitoring have been investigated [16]. Among these, an excellent monitoring method for detecting actions in the home environment called NILM has been established [17,18] and used in the present study. For this study, NILM segments a sum of electrical signals, matches them to appliance signatures by processing with a Factorial Hidden Markov Model, and outputs the estimate of the electrical signal of each appliance. Our NILM technique has high performance in classifying appliances using artificial intelligence and collected power consumption data (Table A1). This NILM technique was registered as an international patent, which estimates the activities of residents efficiently at a low cost. In this study, we estimated habitual behavior of people in their homes using this NILM technology mentioned above.

## 3. Materials and Methods

### 3.1. Study Design and Participants

This prospective observational study used a cohort design based on data from community-dwelling older adults aged ≥65 years living in Nobeoka City, Miyazaki Prefecture, Japan, between April 2019 and July 2020 for over a 1-year period. Participants were recruited through a briefing session, received explanations, provided written informed consent, and underwent interviews and cognitive assessment. Individuals who were moderately or severely demented (MMSE score ≤ 21) were excluded. A total of 94 participants were enrolled in this study. Each participant was followed up for nearly 1 year.

The present study was conducted in accordance with the Declaration of Helsinki and Good Clinical Practice Guidelines. The research protocol was approved by the Ethics Committee of the National Cerebral and Cardiovascular Center (#M30-174-2). All participants provided written informed consent.

### 3.2. Cognitive Assessment

Participants were assessed at baseline using the Japanese version of the Mini-mental State Examination (MMSE) by trained research staff [19]. MMSE is a widely used cognition screening test [20]. It has a maximum score of 30 points and assesses the following five areas of cognitive function: orientation, registration, attention and calculation, word recall, and language. We defined cognitive impairment (Cog) as an MMSE score ≤ 27 and no cognitive impairment (NC) as an MMSE score > 27, based on reports by Kaufer et al. [21] and Damian et al. [22]. Damian et al. reported that this cut-off score discriminated best between normal cognition and cognitive impairment compared to commonly used lower threshold scores [22].

### 3.3. Other Clinical Data

Participants were clinically assessed at baseline using a questionnaire consisting of the following variables: age, sex, years of education (≤9 years or >9 years), drinking status (everyday drinker or not), cigarette smoking (current smoker or other), living situation (living alone or with others), and past and current medical histories (diabetes mellitus, hyperlipidemia, hypertension, stroke, cancer, myocardial infarction, and depressive symptoms). Depressive symptoms were assessed using the 15-item Geriatric Depression Scale (GDS-15) [23], which is suitable for screening depression in community-dwelling older adults. A cutoff point of 4/5 was used to define the presence of depressive symptoms [24]. The number of years of education refers to the participants’ educational background. For example, in the case of a high school graduate, it is calculated as 12 years. In this study, the number of years of education was collected as a continuous value and used as a binary variable of whether it was 9 years or less when constructing prediction models. The reason for this is that less than 9 years of education is often defined as low education. According to a population-based study by Kivipelto et al., people with 7–9 years of education have a 2.5 times higher risk of dementia, and those with 0–6 years have a 3.6 times higher risk of dementia than those with more than 10 years of education [25]. According to the brain reserve hypothesis, those with higher education develop dementia symptoms only with greater pathological changes due to their greater brain capacity [26].

### 3.4. Experimental Setup

Daily activity data were collected using a well-established unobtrusive in-house power monitoring system installed in the homes of participants. The in-house power monitoring technology was developed by Informetis Co., Ltd. and adopted by TEPCO Power Grid, Inc., Tokyo, Japan. Electrical contractors installed a power monitoring sensor on the power distribution board and a mobile router in the living room. Measured information of all power monitoring sensors was acquired via a wireless network of mobile routers, and data were transmitted to the research server via a secure 4G line. In-house power monitoring data were sent from the distributor to the research server in 5-min intervals and were deleted from the device after the data were held for 1 h or more. The sensor was continuously monitored from the date of sensor installation (baseline) until 31 July 2020, and power monitoring data sorted in chronological order were accumulated in the research server owned by TEPCO.

### 3.5. Measurement System for Home Appliance Usage Time

Several electric appliances (air conditioner, microwave oven, washing machine, rice cooker, television (TV), and induction heating (IH)) were monitored. The use of these electric appliances requires a certain degree of cognitive ability and could reflect the daily life pattern of participants. In order to obtain information on the power usage status of each electric appliance, we applied NILM technology.

**Figure 1:**
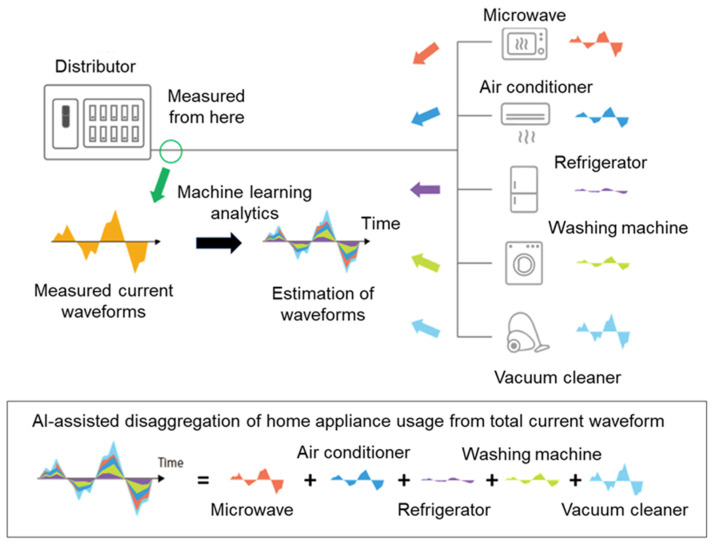
Schematic of NILM technology. NILM technology used in the present study requires only a single sensor installed on the power distribution board, which collects aggregated power consumption data from various appliances, and then analyzes the data using Machine Learning algorithms to produce estimated disaggregation into major home appliance usage from the total current waveform regardless of the manufacturer or model of the home appliance.

**Figure 2:**
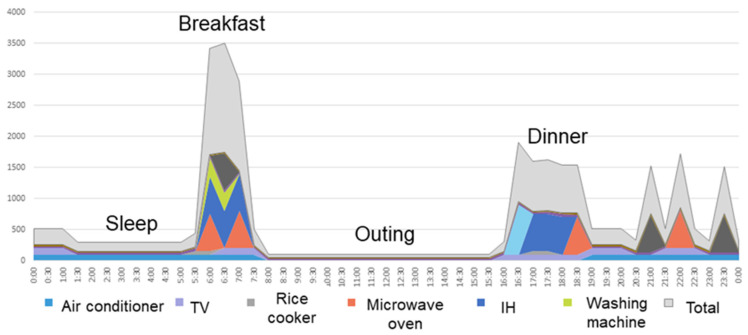
An example of estimated behavior of residents by NILM technology. This technology provides insight into various activities at home via estimated usage time of major home appliances.

NILM technology is a process for providing estimated energy usage by type of major home appliance, including the aforementioned six electric appliances regardless of the manufacturer or model of the home appliance, based on electrical load signatures at a single point in the installation. This technology provides insight into various activities at home via estimated usage of major home appliances (Figure 1 and Figure 2). Further technical details are available in the disclosed patent documentation (No. JP5668204B2, JP5669051B2, JP6135962B2, JP6219401B2, EP2831758A2; “JP” for Japanese patents, and “EP” for European patents). NILM technology used in the present study requires only a single sensor installed on the power distribution board, which collects aggregated current waveform data from various appliances in chronological order, and then analyzes the data using Machine Learning algorithms, such as Factorial Hidden Markov Models, to produce an estimation of each waveform factor of major home appliances. Furthermore, by adopting a power signal coding technology that measures the amplitude and phase of the fundamental and harmonics of the current waveform, it enables a higher-accuracy analysis.

NILM technology has specific advantages related to data quality and assessment, which make it suitable for use in the present study. First, the performance of NILM technology in detecting electric appliance usage has been validated through comparisons of prediction results by NILM technology with actual usage by sensors installed in each home appliance, of which F scores (a performance indicator) were as follows: air conditioner, 0.88; microwave oven, 0.96; washing machine, 0.88; rice cooker, 0.89; IH, 0.90; and TV, 0.69. Other performance indicators of NILM technology are shown in Table A1. Second, TEPCO monitored and checked data quality throughout the study period.

As a pre-processing step, data on electric appliance usage time in each house were converted from seconds to days by summing the usage time of each day.

### 3.6. Statistical Analyses

Baseline characteristics are presented as medians (IQRs) for continuous variables and N (%) for categorical variables. All statistical significance tests were two-sided, using *p* < 0.05 as the level of statistical significance. Generalized linear mixed models (GLMMs) were fitted using “lme4” package [27] and “emmeans” package of R statistical software version 4.0.3 [28]. Statistical analyses and prediction model development were performed with R “caret” package (version 6.0) [29].

Associations between cognitive status and usage time of each home appliance were analyzed with GLMMs, assuming a normal distribution with subject-specific random intercepts by estimating regression coefficients and 95% confidence intervals (CIs). In GLMMs, the usage time of each electric appliance per day was modeled as the dependent variable, with cognitive status (Cog/NC), season, and the interaction term between cognitive status and season as exposure variables. Multivariable-adjusted coefficients were adjusted for days from baseline and season. Based on the GLMMs, estimated marginal means (EMMs) were calculated by cognitive status and season. Mean differences in EMMs were tested for statistical significance.

Two prediction models were developed to identify the cognitive status class (i.e., Cog or NC) of participants. In the present study, a generalized linear model (GLM) was used, assuming a binomial distribution. Model 1 included age, sex, and years of education as possible predictor variables, as they are known to be important risk factors of dementia [30,31]. Model 2 included seasonal average usage times of air conditioner, microwave oven, and IH, in addition to the predictor variables included in Model 1. Reasons for selecting these three electric appliances were as follows. First, we focused on in-home electric appliances that are used every day, excluding those with a fixed one-time operation usage time. Second, we selected home appliances for which usage time differed by cognitive status in the GLMM analyses (i.e., seasonal average usage times of air conditioner, microwave oven, and IH). To take into account seasonal changes in home appliance usage, average usage times for all seasons were included as predictor variables. Finally, from all the above-mentioned predictor variables, optimal predictors were selected by recursive feature elimination (RFE) with 5-fold cross-validation (CV) [32] based on the accuracy of each model. RFE is a wrapper-type feature selection which searches a subset of predictors by first training a model with all possible predictors, ranking all possible predictors by their feature importance, selecting the top one to the maximum number of all possible predictors in order of importance, and making an updated model by the selected predictors; these steps are repeated until the best subset of predictors by the least prediction error is found [32].

The performance of each model was evaluated primarily by accuracy, and secondarily by sensitivity, specificity, positive predictive value (PPV), and negative predictive value (NPV) based on a 2 × 2 confusion matrix. Calibration plots were examined by dividing participants into quartiles to show the agreement between predicted probability from the model and observed proportion of cognitive impairment, which is recommended to be reported by Transparent reporting of a multivariable prediction model for individual prognosis or diagnosis (TRIPOD) reporting guideline [33]. Observed and predicted proportions of cognitive impairment in each quartile were compared by the Hosmer-Lemeshow test [34,35]. Its null hypothesis was that the observed and expected proportions are the same across all quartiles.

## 4. Results

### 4.1. Participants

A total of 94 people participated in this study. Participants were interviewed by researchers and had the in-house power monitoring system installed in their homes. Following baseline evaluation, data were obtained from 78 participants without sensor failure or consent withdrawal. Demographic and clinical characteristics of this final cohort are summarized in Table 1. There were 23 participants with cognitive impairment (Cog group: median age, 78.0 years) and 55 with normal cognition (NC group: median age, 75.0 years). The majority of participants were male (Cog, 74%; NC, 67%). Median MMSE scores for the NC and Cog groups were 29.0 and 26.0, respectively. In the Cog group, the proportions of drinkers and smokers were high. Comorbidities such as diabetes, hyperlipidemia, hypertension, stroke, and myocardial infarction were more common in the Cog group than in the NC group. There was no significant difference in the rate of depressive symptoms between the Cog and NC groups.

### 4.2. Relationships between Electric Appliance Usage Time and Cognitive Status

**Figure 3:**
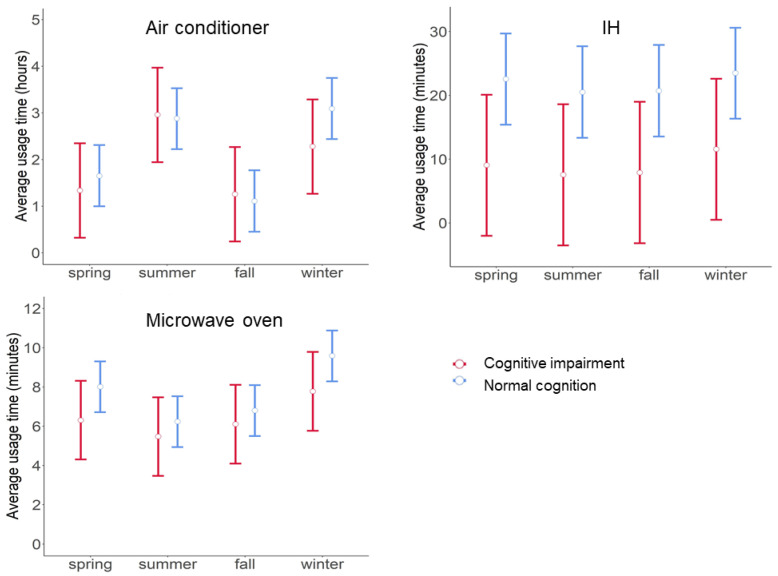
Estimated marginal means (EMMs) of average usage time per day of three in-home appliances in each season by cognitive status. EMMs ± 95% confidence intervals of average usage time per day in each season are plotted for the two groups (i.e., participants with cognitive impairment and those with normal cognition) based on the results of generalized linear mixed models.

We performed GLMM analyses to investigate relationships between the usage time of each appliance and cognitive status. EMMs were calculated for three in-home appliances (Figure 3). The Cog group had shorter usage times of IH, and tended to have shorter usage times of microwave oven only in the spring and the winter and air conditioner only in the winter, compared to the NC group. EMMs of IH usage time were lower in the Cog group compared to the NC group, with differences of −13.5 (95% CI, −26.7–−0.34, *p* = 0.04) in the spring; −13.0 (95% CI, −26.1–0.22, *p* = 0.05) in the summer; −12.8 (95% CI, −26.0–0.37, *p* = 0.06) in the fall; and −11.9 (95% CI, −25.1–1.25, *p* = 0.08) in the winter. EMMs of microwave oven usage time tended to be lower in the Cog group compared to the NC group, with differences of −1.70 (95% CI, −4.09–0.69, *p* = 0.16) in the spring; and −1.80 (95% CI, −4.19–0.58, *p* = 0.14) in the winter. EMMs of air conditioner usage time tended to be lower in the Cog group compared to the NC group, with differences of −0.82 (95% CI, −2.02–0.39, *p* = 0.18) in the winter. We observed no significant differences in EMMs of air conditioner usage time; *p* = 0.60 in the spring; *p* = 0.89 in the summer; and *p* = 0.81 in the fall. We observed no significant differences in EMMs of microwave oven usage time; *p* = 0.53 in the summer; and *p* = 0.57 in the fall.

### 4.3. Prediction Models for Cognitive Impairment with and without In-House Power Monitoring Data

**Figure 4:**
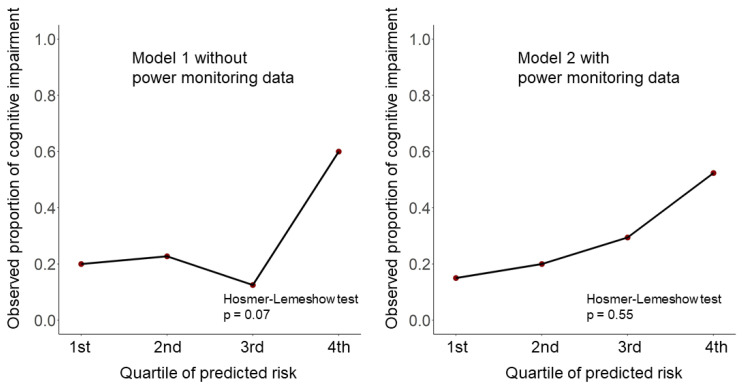
Calibration plots and Hosmer–Lemeshow test values for the two prediction models.

We developed two prediction models to differentiate between people with and without cognitive impairment. As shown in Table 2, the accuracy, sensitivity, and specificity of Model 1 were 0.76, 0.30, and 0.95, respectively. After adding in-house power monitoring data to Model 1, Model 2 had an accuracy of 0.82, sensitivity of 0.48, and specificity of 0.96. The accuracy, sensitivity, and specificity were higher in Model 2 than in Model 1. In Model 1, age and years of education were selected as predictor variables by RFE. In Model 2 (i.e., with average usage times of electric appliances), the following variables were selected by RFE: age, years of education, average usage times of IH in the summer and spring, and the average usage time of air conditioner in the winter. Figure 4 shows the observed proportion of cognitive impairment in each quartile of predicted probability by calibration plots. Both Model 1 without power monitoring data and Model 2 with power monitoring data presented good calibration (Hosmer–Lemeshow test Model 1, *p* = 0.07; Model 2, *p* = 0.55).

## 5. Discussion

This study examined whether electric appliance usage time obtained from a single sensor installed on the power distribution board could be used to identify residents with cognitive impairment. Three independent power monitoring parameters that represent activity behavior (i.e., seasonal average usage times of IH, microwave oven, and air conditioner over 24 h) were found to be associated with cognitive impairment. We also developed prediction models for cognitive impairment using in-house power monitoring data and demographic data. The predictive model that included seasonal average usage times of IH and air conditioner, which were obtained using NILM technology, showed better predictive ability compared to the predictive model that did not include in-house power monitoring data. To the best of our knowledge, this study is the first to report meaningful associations between cognitive function and in-house power monitoring data obtained using NILM technology.

The daily usage times of IH, microwave oven, and air conditioner were associated with cognitive function, consistent with previous reports. Participants with cognitive impairment had shorter usage times of IH and microwave oven. This could be explained by diminished motivation and interest (“apathy”), which is one of the most common behavioral and psychological symptoms of dementia (BPSD). Apathy is highly prevalent across different stages of dementia [36]. Another possible explanation is the inability to cook due to a decline in the ability to perform instrumental activities of daily living (IADL) [37]. In the present study, participants with cognitive impairment had a shorter air conditioner usage time in the winter. This result was in line with a previous report suggesting that blunted responsiveness to temperature is a symptom of dementia [38]. In addition, it is a new lifestyle trend to use air conditioners instead of heaters, and people with dementia have difficulty adapting to the use of new equipment. Alberdi et al. [39] reported that cognitive impairment could be predicted from unobtrusively collected data on daily routine activities (e.g., cooking, eating). In previous studies, the use of in-home monitoring technologies to monitor everyday life activities, such as sleep, mobility, and ADLs, led to the early detection of cognitive impairment [5,7,8]. While functional performance in daily life is an important factor in the diagnosis of dementia, the necessary information can only be collected from patients or their family/relatives. Our predictive models using in-house power monitoring data and demographic data are screening tools for early detection of residents with cognitive impairment, and the definite diagnosis must be confirmed by physicians.

The predictive model developed in this study included a small number of key clinical variables (age, sex, and years of education) [30,31] and power monitoring variables measured by only one sensor, and showed good predictive performance. Interestingly, the addition of power monitoring variables improved the predictive performance of the model, as compared to the model without those variables. The GLM with in-house power monitoring data and clinical data effectively detected cognitive impairment, with an accuracy of 0.82 (95% CI, 0.72–0.90). Reports on prediction models have been scarce. Akl et al. proposed a method of using unobtrusive sensor technologies for home-based automatic detection of cognitive impairment through continuous monitoring [40]. Using sensor data, they were able to automatically detect MCI with an F0.5 score of 0.856, which was calculated with a lower weight of precision than F score, and precision was not reported in this paper. Alberdi et al. reported that cognitive symptoms can be predicted from in-home monitoring data (F score: Random Forest, 0.77; Support Vector Machine, 0.77) [39]. In that study, more than 30 sensors of five types were installed in smart homes. Although their prediction models showed good predictive performance, the installation, management, and evaluation of many sensors were problematic. Additionally, costs associated with the equipment, as well as installation, were an obstacle to introducing the smart environment. Compared to previous studies on in-house monitoring which required numerous sensors to be installed and managed to estimate the overall behavior of residents, the greatest advantage of our system is that it requires only a single sensor installed on the power distribution board, which can be easily implemented to capture various daily activities.

In this study, we included older adults living alone, as well as those living with their families. The reasons for this are as follows. First, if there is a person with cognitive decline in a family living together, the life pattern of the whole family should be affected. Dementia, with its progressive cognitive and functional decline and associated neuropsychiatric symptoms, places a large burden on caregivers [41,42]. Therefore, life patterns can differ between a family with normal cognitive function and a family caring for people with impaired cognitive function. Second, our prediction models for cognitive impairment consider unique values of each individual, by including age, sex, and educational level, although the values of electric appliance usage time variables are the same among a family living together. Third, in the real-world, the proportion of people with cognitive decline who do not live alone is higher than those who live alone [43]. The reason may be that a certain degree of cognitive function is required to perform daily activities necessary to live independently.

This study has several limitations. First, the behavioral data detected by our sensor were not on an individual basis but on a home basis, reflecting the behaviors of all people living in the home including other family members and long-term visitors. NILM technology could not identify the individual user of electric appliances. To obtain accurate data to confirm our results, a future study should focus on people living alone. Second, given the small sample size of the present study, further investigation with a larger number of participants is warranted. Third, this study excluded people with severe dementia. As there is currently no cure for dementia and mild cognitive impairment is considered reversible, the present prediction model was developed as a screening tool for the early stages of cognitive impairment. Further studies are needed to examine the suitability of this predictive model for severe dementia subjects. Fourth, an external validation study should be conducted in the future, although the lack of external validation appears to be a common issue for other existing risk prediction models based on in-house monitoring.

The strength of this study is that it demonstrated the potential of in-house power monitoring to predict and diagnose cognitive impairment at an early stage by allowing for continuous monitoring and detection of changes in the daily life of older adults. In addition, unlike other in-house sensors, our sensor is installed in one place (i.e., on the distribution board). As there is no need to attach multimodal sensors in various places, our system can be easily adapted to any house. In addition, the NILM technology used in this study has been registered as an international patent (EP2831758A2), and it is a technology that can separate an aggregated current waveform into individual waveforms of major home appliances, even if the manufacturer and model of the home appliance are different. Therefore, this NILM technology can be implemented in the real world without the need to build a laboratory setting.

In consideration of the aforementioned limitations, we offer recommendations for future studies. First, we constructed prediction models using average power data of each home appliance on a seasonal basis. For future application, time series analysis focusing on the change from the past in each individual can be considered. Moreover, by increasing the number of participants and performing analyses in groups with varying cognitive function, AI-based analysis will be possible. This will allow for detection of not only simple linear regression but also relationships including non-linearity, thereby improving the performance of the prediction model. With further testing and validation, the power monitoring parameters measured in this study can be used to facilitate in-house continuous remote monitoring to track cognitive frailty over time. TEPCO aims to develop this in-house power monitoring system into installation as a social infrastructure in the future, which can be provided as a ubiquitous service when needed.

## 6. Conclusions

In-house power monitoring technologies can be used to identify people with cognitive impairment. This study represents the first step toward building a reliable system that integrates real-life power information into a potentially useful automated warning tool for the early detection of and medical intervention for cognitive impairment.

## Figures and Tables

**Table 1:** Participant baseline demographics and clinical characteristics.

	MMSE > 27 Normal Cognition	MMSE ≤ 27 Cognitive Impairment	Total
	(N = 55)	(N = 23)	(N = 78)
Continuous variables, median (IQRs)			
Age	75.0 (70.0, 78.5)	78.0 (75.0, 83.5)	76.0 (70.0, 80.0)
Men	37 (67.3%)	17 (73.9%)	54 (69.2%)
MMSE	29.0 (29.0, 30.0)	26.0 (24.0, 27.0)	29.0 (27.0, 30.0)
Years of education	12.0 (11.5, 12.5)	12.0 (10.5, 12.5)	12.0 (11.0, 12.8)
Usage hours of air conditioner	1.59 (0.44, 3.26)	1.67 (0.31, 3.32)	1.63 (0.35, 3.33)
Usage hours of microwave oven	0.12 (0.06, 0.17)	0.10 (0.04, 0.14)	0.115 (0.06, 0.17)
Usage hours of IH	0.15 (0.001, 0.71)	0.00 (0, 0.12)	0.02 (0, 0.56)
Categorical variables, *n* (%)			
Everyday drinker	21 (38.2%)	10 (43.5%)	31 (39.7%)
Current smoker	3 (5.5%)	2 (8.7%)	5 (6.4%)
Living alone	15 (27.3%)	3 (13.0%)	18 (23.1%)
Medical history			
Diabetes mellitus	5 (9.1%)	3 (13.0%)	8 (10.3%)
Hyperlipidemia	16 (29.1%)	11 (47.8%)	27 (34.6%)
Hypertension	23 (41.8%)	11 (47.8%)	34 (43.6%)
Stroke	2 (3.6%)	3 (13.0%)	5 (6.4%)
Cancer	10 (18.2%)	4 (17.4%)	14 (17.9%)
Myocardial infarction	3 (5.5%)	4 (17.4%)	7 (9.0%)
Depressive symptoms	13 (23.6%)	3 (13.0%)	16 (20.5%)

Abbreviations: MMSE, Mini-Mental State Examination; and IQR, interquartile range.

**Table 2:** Prediction performance of models for cognitive impairment with and without in-house power monitoring data.

Model	Model 1 without Power Monitoring Data	Model 2 with Power Monitoring Data
Accuracy	0.76 (0.65–0.85)	0.82 (0.72–0.90)
Sensitivity	0.30 (0.13–0.53)	0.48 (0.27–0.69)
Specificity	0.95 (0.85–0.99)	0.96 (0.87–1.00)
Positive predictive value	0.70 (0.35–0.93)	0.85 (0.55–0.98)
Negative predictive value	0.76 (0.65–0.86)	0.82 (0.70–0.90)

## Data Availability

Restrictions apply to the availability of data. Data were obtained from TEPCO and are not publicly available.

## Appendix A

**Table A1:** Performance of NILM technology for each electric appliance when using testing datasets, based on information from Informetis Co., Ltd.

	F Score	Precision	Recall	Specificity	Negative Predictive Value	Cohen Kappa	Accuracy
Air conditioner	0.88	0.91	0.90	0.94	0.91	0.80	0.95
Microwave oven	0.96	0.97	0.95	1.00	1.00	0.96	1.00
Washing machine	0.88	0.87	0.90	1.00	1.00	0.88	0.99
Rice cooker	0.89	0.94	0.85	1.00	1.00	0.89	1.00
IH	0.90	0.92	0.91	0.99	1.00	0.89	0.99
TV	0.69	0.81	0.68	0.91	0.83	0.59	0.83

Abbreviations: IH, induction heating; TV, television.
